# Efficient Mn(II) removal mechanism by *Serratia marcescens* QZB-1 at high manganese concentration

**DOI:** 10.3389/fmicb.2023.1150849

**Published:** 2023-04-27

**Authors:** Xuejiao Huang, Xiaofang Nong, Kang Liang, Pengling Chen, Yi Zhao, Daihua Jiang, Jianhua Xiong

**Affiliations:** ^1^State Key Laboratory for Conservation and Utilization of Subtropical Agro-bioresources, Guangxi University, Nanning, Guangxi, China; ^2^Guangxi Bossco Environmental Protection Technology Co., Ltd., Nanning, China; ^3^School of Resources, Environment and Materials, Guangxi University, Nanning, Guangxi, China

**Keywords:** *Serratia marcescens*, manganese, oxidation, oxidation-related enzyme activity, adsorption

## Abstract

Manganese (Mn(II)) pollution has recently increased and requires efficient remediation. In this study, *Serratia marcescens* QZB-1, isolated from acidic red soil, exhibited high tolerance against Mn(II) (up to 364 mM). Strain QZB-1 removed a total of 98.4% of 18 mM Mn(II), with an adsorption rate of 71.4% and oxidation rate of 28.6% after incubation for 48 h. The strain synthesized more protein (PN) to absorb Mn(II) when stimulated with Mn(II). The pH value of the cultural medium continuously increased during the Mn(II) removal process. The product crystal composition (mainly MnO_2_ and MnCO_3_), Mn-O functional group, and element-level fluctuations confirmed Mn oxidation. Overall, strain QZB-1 efficiently removed high concentration of Mn(II) mainly via adsorption and showed great potential for manganese wastewater removal.

## 1. Introduction

Manganese (Mn) is an important trace element for all living organisms. However, excessive amounts of Mn can cause severe damage to plants, animals, and humans (Neculita and Rosa, 2019; Wu et al., 2022). In recent years, the over-exploitation of Mn ores and its widespread use in the steel industry have resulted in large discharges of Mn into surface waters, causing significant environmental pollution (Huang et al., 2020; Wu et al., 2022). Soluble Mn(II) is the main form of Mn and is toxic, making its treatment crucial in Mn-polluted wastewater (Tang et al., 2019). The concentration of Mn(II) in electrolytic manganese wastewater has been reported to reach 35.7 mM, with some even reaching a peak value of 268 mM, which is much higher than the World Health Organization’s standard Mn content for drinking water (<0.002 mM) (Xu et al., 2014; Tang et al., 2019). Therefore, suitable methods for removing Mn(II) are urgently needed.

Traditional methods for removing heavy metals include chemical oxidation, adsorption, and precipitation, which typically provide high removal efficiencies, but have the disadvantages of being expensive, and might potentially produce toxic by-products (Huang et al., 2018; Wang et al., 2019; Shrestha et al., 2021). In comparison, bioremediation is a low-cost technology with a simple operation procedure that can quickly and effectively remove heavy metals while not affecting the physicochemical properties of the environment (Abinandan et al., 2019; Giovanella et al., 2020). Research has shown that microorganisms that survive long-term in heavy-metal-contaminated environments can resist the stress induced by these metals. Moreover, these microorganisms can reduce the heavy metal content through surface adsorption, extracellular precipitation, and intracellular detoxification, potentially allowing pollution remediation (Mohapatra et al., 2019; Al-Ansari et al., 2021; Pramanik et al., 2021).

Some Mn(II)-tolerant bacteria known to effectively remove Mn(II) have been reported in the genera *Bacillus*, *Pseudomonas*, *Pedomicrobium*, *Gallionella*, *Leptothrix*, and *Aminobacter* (Zhang et al., 2002; Tebo et al., 2004; Tang et al., 2016; Wan et al., 2017; Zhao et al., 2019; Therdkiattikul et al., 2020; Tie et al., 2022). Biogenic Mn(II) removal has broad prospects in the remediation of polluted water in developing countries because of its low cost, wide applicability, and environmental benefits. However, most of the reported bacteria with Mn(II) removal capabilities have relatively low Mn(II) tolerance (<100 mM) and removal efficiency (Tang et al., 2016; Wan et al., 2017; Zhao et al., 2019; Therdkiattikul et al., 2020; Tie et al., 2022). Microorganisms typically reduce the toxicity of Mn(II) by adsorbing or oxidizing Mn(II) into insoluble Mn oxide precipitates (Zhang et al., 2019). Microorganism can transform Mn through surface adsorption, direct oxidation, and indirect oxidation (Tie et al., 2022). Enzymes thought to participate in Mn oxidation catalysis include multicopper oxidase, Mn peroxidase, and Mn catalase (Whittaker, 2012; Zeng et al., 2018; Zhao et al., 2023). The Mn(II) removal mechanisms of various microorganisms are complex and require further exploration.

Based on the limited research on microorganisms with high Mn(II) tolerance and Mn(II) removal efficiency, this study isolated a strain highly tolerant to Mn(II) (up to 364 mM), *Serratia marcescens* QZB-1. The objectives of this study were to explore (i) the influence of different factors on Mn(II) removal in strain QZB-1; (ii) its adsorptive and oxidative activity during the Mn(II) removal process; and (iii) the underlying mechanism of Mn(II) removal.

## 2. Materials and methods

### 2.1. Microorganism and culture medium

*S. marcescens* QZB-1 (Genbank No. MZ182358), isolated from acidic red soil (Wang M. S. et al., 2022), was used in this study. The Luria–Bertani (LB) medium used for bacterial enrichment contained 10 g tryptone, 10 g NaCl, and 5 g yeast extract per liter. The sterilized LB medium (121°C, 30 min) was cooled to 30°C, and MnCl_2_ was then filtered through a 0.45 μm microporous membrane to prepare the medium containing different concentrations of Mn(II).

### 2.2. Investigation of the Mn(II) tolerance ability of strain QZB-1

Strain QZB-1, which was precultured in LB medium (100 mL, pH 7.1–7.2), was inoculated into an LB medium (100 mL, pH 5.5) with 0–364 mM of Mn (II) (with the initial OD_600_ maintained around 0.1). A control test was also performed without inoculation. The cultures were incubated at 30°C and 150 rpm for 48 h. All experiments were conducted in triplicate. Samples were taken from the cultures to determine the OD_600_ and the level of Mn(II).

### 2.3. Mn(II) removal by strain QZB-1

The pure strain QZB-1 was inoculated into LB medium containing 18 mM Mn(II) to investigate the impact of temperature, initial pH, and agitation on QZB-1-driven Mn(II) removal (with the initial OD_600_ maintained around 0.1). During the pH experiments, the initial pH was adjusted to 3, 4, 5, 5.5, or 6 using NaOH and HCl. In temperature experiments, the temperature was set to 10, 20, 30, 35, or 40°C. In the agitation experiments, the shaking speed was set to 0, 50, 100, 150, or 200 rpm, as per previous studies (Chen et al., 2021; Huang et al., 2021; Yan et al., 2021). The cultures were incubated for 2 days, and samples were then collected to measure the pH, OD_600_, and Mn(II) concentration.

The removal route of Mn(II) by strain QZB-1 was further explored. The precultured cells were inoculated in an LB medium (pH 5.5) with 18 mM Mn(II) (the initial OD_600_ maintained around 0.1) and cultured at 150 rpm and 30°C for 48 h. The culture medium without bacterial cells was used as a negative blank control. Aliquots of the bacterial suspension were periodically collected to determine the different forms of Mn. Briefly, a 5 ml bacterial suspension was centrifuged at 8000 × *g* and 4°C for 10 min. The supernatant was filtered through a 0.45 μm filter to measure the residual Mn(II) (soluble Mn) in the solution. The obtained bacterial cell pellets were redissolved with an equal volume of 50 mM CuSO_4_ solution, vibrated overnight, and then centrifuged at 8000× *g* and 4°C for 10 min to obtain the supernatant containing adsorbed Mn. The precipitate was treated with 20 mM hydroxylamine hydrochloride for more than 10 h, then centrifuged at 8000× *g* and 4°C for 10 min to obtain the oxidized Mn (Wan et al., 2017).

### 2.4. Extracellular polymeric substance analysis

To understand the mechanism of QZB-1-driven Mn(II) removal, the extracellular polymeric substances (EPS) produced during the growth of the strain were analyzed. Strain QZB-1 was inoculated in LB medium with or without 18 mM Mn(II). Samples were periodically collected to extract the EPS. The extraction of EPS was based on the method by Shi et al. (2020). The proteins (PN) and polysaccharides (PS) in the EPS were analyzed using the Lowry procedure and phenol-sulfuric acid method, respectively (Shi et al., 2020).

### 2.5. Oxidation factor location analysis

Strains were inoculated in an LB medium and then cultured at 30°C and 150 rpm for 72 h. The bacterial suspension was centrifuged at 2000 × *g* for 10 min to obtain the liquid supernatant (10 mL, marked as A). The culture was centrifuged at 600× *g* for 2 min, and the obtained bacterial pellets were washed with 1 M Tris–HCl and resuspended in 10 mL Tris–HCl to prepare the bacterial resuspension (marked as B). The bacterial resuspension was dispersed by ultra-sonication for 10 min, then 10 mL of supernatant was sampled as the bacterial lysate (marked as C) after being centrifuged at 1000× *g* for 10 min (Bai et al., 2021).

Subsequently, Mn(II) solution was added to the above-prepared liquid (10 ml) (the final Mn(II) concentration was 18 mM). The culture was shaken at 150 rpm and 30°C for 2 h. The Mn (II) content in the supernatant was determined after the culture was centrifuged at 2000× *g* for 10 min.

### 2.6. Spectroscopic Mn(III) trapping experiment

To investigate the Mn(III) intermediates produced during Mn oxidation by strain QZB-1, sodium pyrophosphate (PP) was selected as the trapping reagent to capture Mn(III) (Wan et al., 2017; Bai et al., 2020, 2021). The isolate QZB-1 was inoculated into an LB medium supplied with 18 mM Mn(II) and cultured at 150 rpm and 30°C for 96 h. The culture medium without Mn(II) or bacterial cells was used as a positive and negative blank control, respectively. Ten milliliters of the above three media were periodically added to 1 ml of 10 mM PP solution and reacted at 30°C. The Mn(III)-sodium pyrophosphate complex was detected by spectrophotometry at wavelengths of 200–600 nm after filtering the reaction solution through a 0.22 μm microporous membrane.

### 2.7. Determination of Mn oxidation-related enzyme activity of the strain

Strain QZB-1 was inoculated in an LB medium containing 18 mM Mn(II) at 150 rpm and 30°C for 48 h. The cultures were centrifuged at 3000× *g* for 2 min at 4°C. The obtained bacterial pellets were washed twice with 50 mM Tris–HCl buffer solution (pH 8.0), resuspended in the same buffer, disrupted by an ultrasonic cell disruptor for 15 min, and then centrifuged at 3000× *g* for 2 min to obtain the supernatant as a crude enzyme liquid. The reaction mixture (3 ml) for multiple copper oxidase activity determination contained 2.9 ml sodium citrate buffer, containing 0.5 mM 2,2′-biazo-bis-3-ethylbenzothiazoline-6-sulfonic acid (ABTS), 1 mM CuCl_2_, and 0.1 mL crude enzyme (pH 5.0). The amount of enzyme required for the oxidation of 1 μmoL ABTS per minute was defined as one enzyme activity unit (U) (Yang et al., 2017). In the Mn peroxidase (MnP) activity assay, the reaction mixture (3.2 mL) contained 2 mL 100 mM succinic acid buffer, 0.7 mL 4 mM guaiacol solution, 0.2 mL 5 mM MnSO_4_ solution, 0.1 mL 2.5 mM H_2_O_2_ solution, and 0.2 mL crude enzyme solution (pH 5.5). The MnP enzyme activity was defined as one enzyme activity unit (U) required to catalyze 1 nM guaiacol to tetroxylenol per milligram of protein per minute (Cui et al., 2020). The reaction mixtures for the Mn catalase (CAT) activity assay consisted of a 0.1 mL enzyme solution sample and 2.9 mL 50 mM TriS–HCl buffer (pH 8.0) containing 10 mM H_2_O_2_. The unit of enzyme activity (U/mL) was defined as the amount of CAT enzyme required to decompose 1 μmol H_2_O_2_ per minute at 37°C and pH 5.5 (Ebara and Shigemori, 2008).

### 2.8. Characterization of biological Mn oxide

Strain QZB-1 was cultured in an LB medium with or without 18 mM Mn (II) for 48 h, then centrifuged at 3000× *g* for 10 min to obtain pellets. For scanning electron microscopy and energy dispersive (SEM-EDS) analysis, the pellets were fixed with 2.5% glutaraldehyde at 4°C for 24 h and then dehydrated in ethanol solutions (30, 50, 70, 90, and 100%) before drying. For X-ray photoelectron spectroscopy (XPS), X-ray diffraction (XRD), and Fourier transformed infrared (FTIR) analyses, the pellets were dried using an ultra-low temperature freezer (ALPHAL-4LD PLUS, CHRIST Co., Germany) (Zhang et al., 2019; Huang et al., 2022). SEM-EDS, XPS, XRD, and FTIR analyses were conducted using S-4800 scanning electron microscope and energy spectrum analyzer, X-ray photoelectron spectroscopy (ESCALAB 250XI+, Thermo Fisher Scientific, USA), D/Max-3C X-ray diffractometer with Cu-K radiation, and FTIR spectrophotometer in the range of 4,000–400 cm^−1^, respectively.

### 2.9. Analytical methods

The OD_600_ was quantified using an automatic growth curve analyzer (BIOSCREEN C, Finland) and a spectrophotometer. The pH value was detected with a pH electrode. The Mn(II) concentration of the supernatant after the sample was centrifuged at 2000× *g* for 5 min was determined by an inductively coupled plasma emission spectrometer (ICP-5000, Beijing Juguang Technology Co. LTD, China).

Microsoft Excel 2010 and SPSS Statistics 22 were used to analyze the data, and graphs were constructed using Origin 8.6 software. All the results are presented as means ± standard deviation (SD) of three replicates. One-way ANOVA was used to determine the statistical significance of all variants and *p* < 0.05 was considered statistically significant.

## 3. Results and discussion

### 3.1. Tolerance ability of isolate QZB-1 against Mn(II)

**Figure 1:**
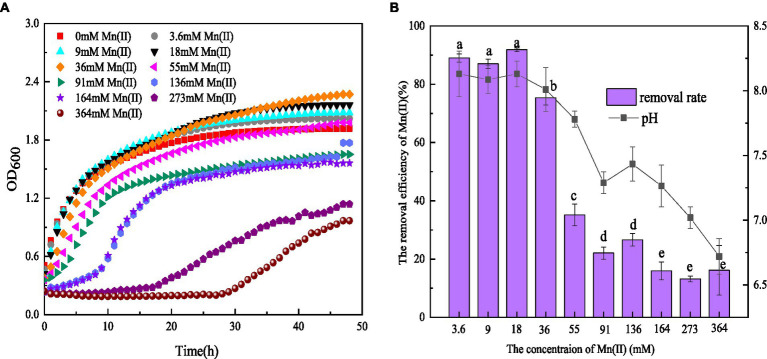
**(A)** Growth and **(B)** Mn(II) removal efficiency of strain QZB-1 incubated in the LB medium with different concentrations of Mn(II) at 30°C and 150 rpm for 48 h.

It was found that 3.6–36 mM Mn(II) could stimulate the growth of strain QZB-1. The growth of QZB-1 was inhibited when the Mn(II) concentration exceeded 55 mM (Figure 1A). However, QZB-1 showed a growth tendency after 28 h of incubation, even in a medium containing 364 mM Mn(II). This illustrated that QZB-1 could tolerate up to 364 mM Mn(II), which was a significantly higher-concentration than that for *Arthrobacter* sp. H1, *Arthrobacter sp*. HW-16, and *Ralstonia pickettii* HM8 (Yan et al., 2014; Wan et al., 2017; Huang et al., 2018). In addition, the Mn(II) removal efficiency by QZB-1 was influenced by the Mn(II) content. Strain QZB-1 could remove more than 80% of Mn (II) when the initial Mn(II) concentration was less than 18 mM. The removal efficiency decreased with an increase in the Mn(II) concentration once the concentration of Mn(II) in the medium was over 18 mM. The highest Mn(II) removal efficiency (91.8%) was obtained when the initial Mn(II) concentration was 18 mM (Figure 1B). The reported bacterial Mn(II) removal predominantly exhibited lower Mn(II) removal efficiency than strain QZB-1. For example, *Acinetobacter* sp. AL-6 could hardly remove Mn(II) in 48 h when the initial Mn(II) reached 3.6 mM (An et al., 2021), and less than 70% of the 18 mM Mn(II) in the medium was removed after incubating *Aminobacter* sp. H1 and *Ralstonia pickettii* HM8 for 120 h (Yan et al., 2014; Huang et al., 2018). To date, most strains of *Serratia sp.* have shown excellent Ni(II), Co(II), Cr (VI), and Pb(II) removal abilities (Han et al., 2018; Díaz et al., 2022; Wang X. Y. et al., 2022). Only a few strains have been reported to have Mn(II) removal capabilities. For instance, *S. marcescens* CL11 and CL35, isolated from Mn mine water, could tolerate 22 mM Mn(II) and remove over 55% of 0.8 mM Mn(II) (Barboza et al., 2017)*. S. marcescens* KH-CC, isolated from rat-hole coal mines, could tolerate 15 mM Mn(II) and remove 72.5% of 1 mM Mn(II) (Shylla et al., 2021). However, the removal mechanism of Mn(II) by *Serratia sp.* remains unclear. In this study, *S. marcescens* QZB-1, isolated from acidic red soil, could grow and remove Mn(II) even at an Mn(II) content of 364 mM (Figure 1). This indicated that *S. marcescens* QZB-1 has an excellent Mn(II) tolerance and removal ability.

pH is one of the major factors affecting Mn oxidation (Barboza et al., 2015; Tang et al., 2019). In this study, the pH values in LB medium without Mn(II) increased from 5.5 to 7.9 after 48 h of cultivation (data not shown), suggesting the release of alkaline substances by strain QZB-1. An Mn(II) concentration ranging from 3.6–36 mM could stimulate the strain to produce alkaline substances, consequently increasing the pH above 8.0 (Figure 1B). These results illustrated that strain QZB-1 could promote Mn(II) oxidation by changing the pH of the medium.

### 3.2. Optimal environmental factors for Mn(II) removal By strain QZB-1

**Figure 2:**
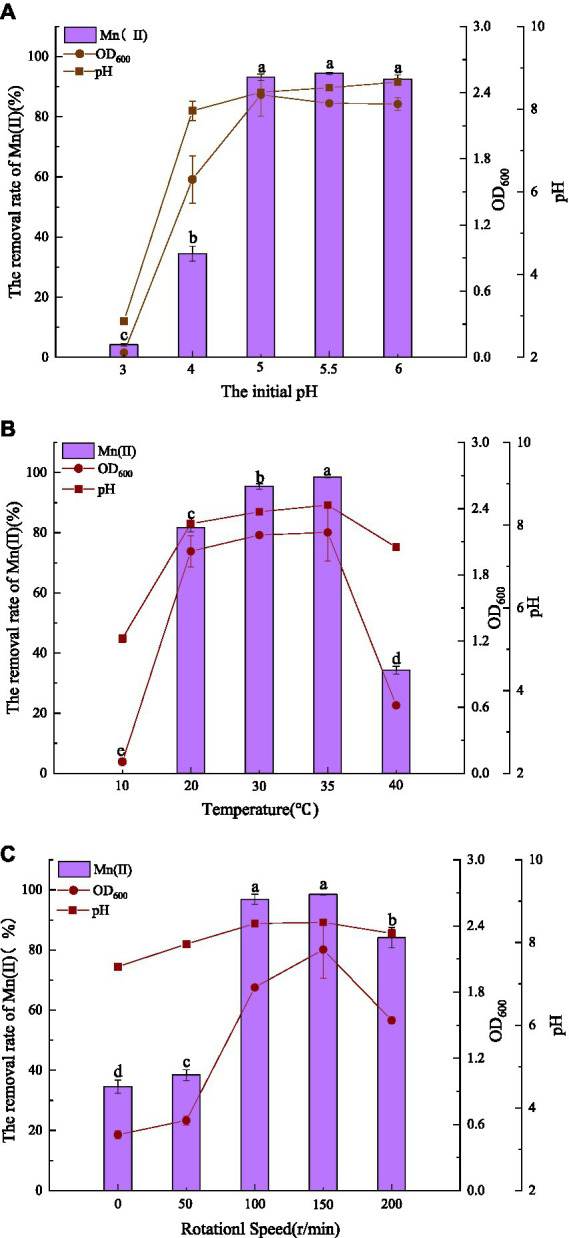
Influence of environmental factors (**A**: initial pH; **B**: temperature; **C**: agitation) on QZB-1 growth and Mn(II) removal efficiency incubation at 30°C and 150 rpm for 48 h.

The effects of pH, temperature, and agitation on Mn(II) removal by QZB-1 are displayed in Figure 2. Mn(II) is oxidized at pH ≥ 7.0; therefore, the pH was set between 3.0–6.0. Strain QZB-1 grew slowly at pH 3.0 and removed only 4.14% of Mn(II) (Figure 2A). With an increase in pH, the growth of the strain accelerated, and the removal rate of Mn(II) also significantly increased. When the pH was 5.0–6.0, the removal rate of Mn(II) reached more than 90%, indicating that the strain could grow under weak acidic conditions and efficiently remove Mn(II). Except for the pH 3.0 medium, the pH in all systems increased to varying degrees during culture, likely due to the release of alkaline substances by the strain. The optimum pH for Mn(II) removal by strain QZB-1 was pH 5.5, which was lower than that of most of the previously reported Mn-oxidizing strains (Miyata et al., 2004; Sasaki et al., 2006; Huang et al., 2018; Zhang et al., 2019).

Figure 2B shows the influence of temperature on Mn removal by the strain at an initial pH of 5.5. Strain QZB-1 could not grow and remove Mn(II) at a low temperature (10°C); however, an increase in temperature enhanced the removal efficiency of Mn(II). Accompanied by the growth of the strain, the removal efficiency reached its maximum (98.4%) at 35°C. When the temperature was above 35°C, strain QZB-1 grew slowly because of high-temperature stress, resulting in a decreased Mn(II) removal rate. The temperature could affect the activity of bacterial enzymes and their Mn(II) removal abilities. Different microbial species have different tolerances to temperature, resulting in different optimal temperatures for Mn (II) removal (Zhao et al., 2018; Tie et al., 2022). Similar to the observation in the pH experiment (Figure 2A), the pH value of the medium increased after culturing.

Strain QZB-1 grew slowly and could remove approximately 40% Mn(II) at 0 and 50 rpm, indicating that the strain QZB-1 maintains its Mn(II) removal ability under micro-anaerobic conditions. With an increase in the rotation speed, the dissolved oxygen content in the culture medium gradually increased, which stimulated the growth of strain QZB-1 and the release of alkaline substances, thereby promoting Mn(II) removal (Figure 2C). The Mn(II) removal efficiency was at its maximum at 150 rpm and then decreased when the rotating speed was higher than 150 rpm. This might be because the growth of bacteria and the capability to remove the contaminant were inhibited when the content of oxygen exceeded a critical level (Lei et al., 2019; Hou et al., 2021; Huang et al., 2021). Overall, strain QZB-1 showed a high tolerance against to Mn(II) (up to 364 mM) and could efficiently remove 18 mM of Mn(II) under the following conditions: pH 5.5, 35°C, and 150 rpm.

### 3.3. Mn(II) removal characteristics by the QZB-1 isolate

**Figure 3:**
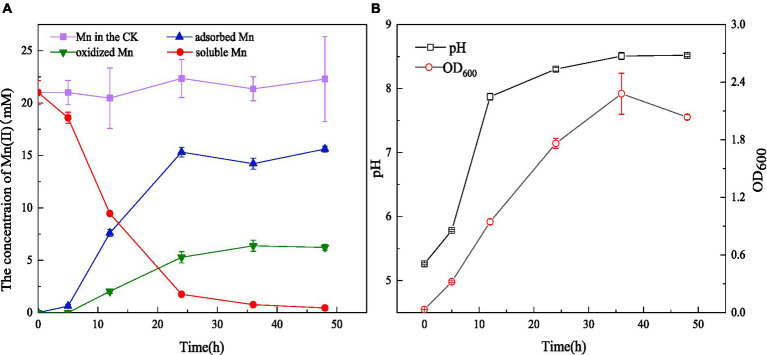
Mn(II) adsorption and oxidation by strain QZB-1 with initial Mn(II) concentration of 18 mM within 48 h **(A)**, changes in OD_600_ and pH during the cultivation process **(B)** (Mn in the CK represents the Mn concentration in the negative blank control).

Strain QZB-1 was cultivated in an 18 mM Mn(II)-containing medium (pH 5.5) at 35°C and 150 rpm. Compared with the control, Mn(II) in the supernatant gradually decreased after culturing QZB-1 (Figure 3A), and the Mn(II) removal rate reached 92.2% after 24 h of incubation. By contrast, only 80% of 18 mM Mn(II) was removed by *Aeromonas hydrophila* DS02 after 144 h cultivation(Zhang et al., 2019), and 59 and 88% of 1 mM Mn(II) was removed by *Lysinibacillus* sp. and *Brevibacillus* spp., respectively, even when cultured for 144 h (Tang et al., 2016; Zhao et al., 2018). The amount of adsorbed and oxidized Mn increased significantly from 6 to 24 h. This was consistent with the results reported by Yan et al. (2014), who found that a decrease in the Mn(II) concentration in the supernatant was accompanied by a noticeable increase in adsorbed and oxidized Mn during 40 h of cultivation of *Aminobacter* sp. However, the amount of adsorbed Mn was greater than that of oxidized Mn after incubation of strain M for 48 h. A similar phenomenon was observed in *Acinetobacter* sp. AL-6 (An et al., 2021) and *Aminobacter* sp. H1 (Yan et al., 2014), while *Aeromonas hydrophila* strain DS02 absorbed and oxidized equal Mn(II) after incubation for 48 h (Zhang et al., 2019). Conversely, the amount of adsorbed Mn was lower than that of oxidized Mn after incubation of *Cupriavidus* sp. HY129 for 48 h. Gupta and Sivakumaran (2016) found that the surface of *E.coli* has more functional groups in the presence of heavy metals, and its adsorption capacity of heavy metals was enhanced. Thus, the high absorption ability of Mn(II) by strain QZB-1 might be because of the abundant functional groups on its surface, which needs further study. After 24 h, the adsorbed and oxidized Mn gradually reached saturation, which may be related to the stable growth period of the strain. The pH gradually increased from 5.5 to 8.5 throughout the whole growth period of strain QZB-1(Figure 3B), which was consistent with the above results.

### 3.4. Mn(II) removal mechanism by the QZB-1 isolate

#### 3.4.1. Morphology and physicochemical characteristics of biogenic Mn oxides (BMO)

**Figure 4:**
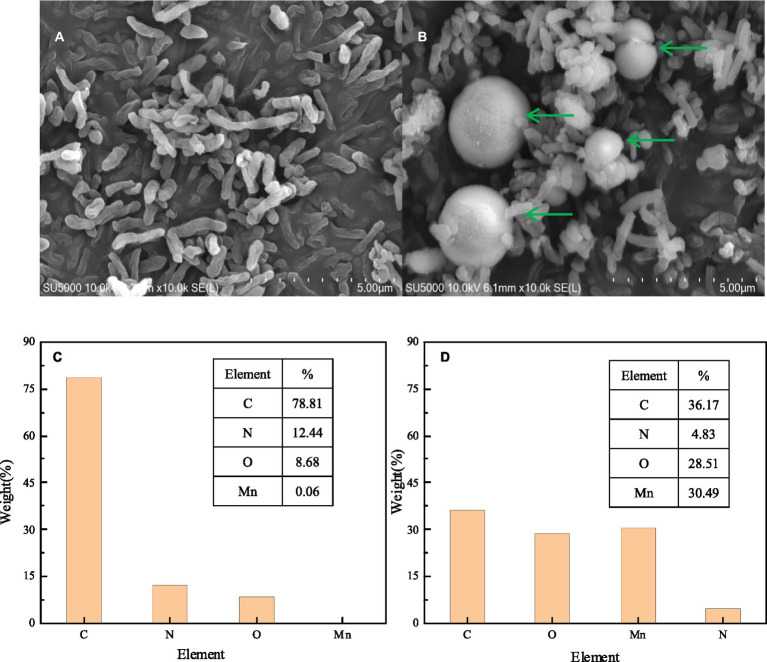
SEM micrograph EDS element map analysis of strain QZB-1 inoculated in LB medium without Mn(II) **(A,C)** and with 18 mM Mn(II) **(B,D)**.

SEM-EDS, FTIR, and XRD analyses were performed to characterize the composition and structure of BMO produced by the Mn oxidation of strain QZB-1. The SEM image of *S. marcescens* QZB-1, cultivated in an LB medium (Figure 4A), revealed short rod cells. Circular BMO aggregates (green arrows in Figure 4B) were found when culturing strain QZB-1 in an LB medium containing 18 mM Mn(II) for 48 h. This was similar to the result of Zhang et al. (2019). There were little Mn elements in cells in an Mn-free environment according to the EDS analysis (Figure 4C). However, the proportion of Mn elements increased to 30.49% after the incubation of strain M in LB medium with 18 mM Mn(II) for 48 h (Figure 4D), which indicated the adsorption and deposition of Mn on the cells. Moreover, the increase in O element content further clarified the formation of BMO.

**Figure 5:**
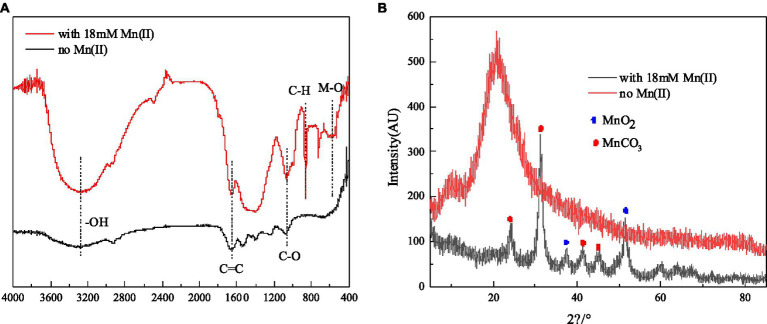
FTIR spectrum **(A)** and XRD **(B)** of strain QZB-1 cultured with 18 mM Mn(II) or without Mn(II).

The results of FTIR analysis of strain QZB-1 cells, incubated with and without Mn(II), are shown in Figure 5B. The band around 3,300 cm^−1^ corresponds to the stretching vibration of -OH group. The band around 1,650 cm^−1^ corresponds to C=C groups, while the band at 1018 cm^−1^ is typical of C-O, which may indicate the presence of polysaccharides (Nkoh et al., 2019). The vibrations of these two bands were enhanced when strain QZB-1 reacted with Mn(II). Moreover, a new peak at 860 cm^−1^ represented the vibration of C-H (An et al., 2021). The results illustrated that functional groups on the surface of the QZB-1, such as polysaccharides, were more abundant and altered in the presence of Mn(II). This explains why strain QZB-1 removed Mn(II) mainly through adsorption. Furthermore, the Mn–O band, which is mainly derived from MnO_2_ or Mn_2_O_3_ (Dessie et al., 2020), was observed at 580 cm^−1^ in this study, indicating the oxidation of Mn(II) during its removal by strain QZB-1.

XRD analysis was performed to identify the phase of the samples of strain QZB-1 cultured in an LB medium with and without Mn(II), and the result is shown in Figure 5A. The diffraction peaks at 2θ of 37.44° and 51.46° were indexed to MnO_2_ (JCPDS-73-1539) (An et al., 2021; Bai et al., 2021), which confirmed the existence of the biological Mn oxidation. The characteristic peaks at 2θ of 24.38°, 31.3°, 41.2°, and 45.3° corresponded to the phases of MnCO_3_ (rhodochrosite, JCPDS 44–1,472) (Li et al., 2020). Rhodochrosite is found to idiomorphically occur at pH 8 under alkaline conditions (Tian et al., 2018). In this study, the pH increased from 5.5 to 8.5 during the experimental process. Thus, the accumulation of rhodochrosite might occur simultaneously through the abiotic transformation of Mn(II) to MnCO_3_.

#### 3.4.2. Clarification of Mn(III) production in the Mn(II) oxidation process

**Figure 6:**
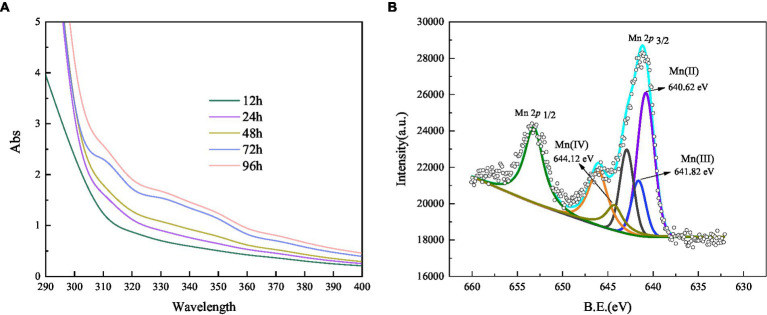
Absorbance value of Mn(III) -pyrophosphate in the bacterium suspension **(A)**, and XPS analysis of biogenic manganese oxides **(B)**.

A trapping experiment using Mn(III)-NaPP as an index for Mn (III)-intermediates was performed (Webb et al., 2005), and the result is shown in Figure 6A. After incubation, the Mn(II) concentration did not decrease in the negative blank control, and there was no Mn(III)-PP production. When the strain was cultivated for 12 h, the spectrum was nearly unchanged, indicating the weak oxidation of Mn(II). A characteristic peak appeared at 310 nm after incubation for 24 h, which corresponded to the Mn(III)-NaPP complex, indicating the presence of Mn(III) during the Mn(II) oxidation by strain QZB-1. The absorption spectra at 310 nm increased initially and then decreased as the bacterial cultivation time was extended (Figure 6A), which was consistent with the Mn(II) oxidation characteristic of strain QZB-1 (Figure 3). Three Mn species (Mn(II), Mn(III), and Mn(IV)) were observed in the substances produced by the reaction of the strain with Mn(II), according to the XPS analysis (Figure 6B; Raj et al., 2010; Zhang et al., 2019). These results demonstrated that the Mn oxidation process of QZB-1 was via Mn(III).

#### 3.4.3. Location for Mn oxidation and key enzyme activity

**Figure 7:**
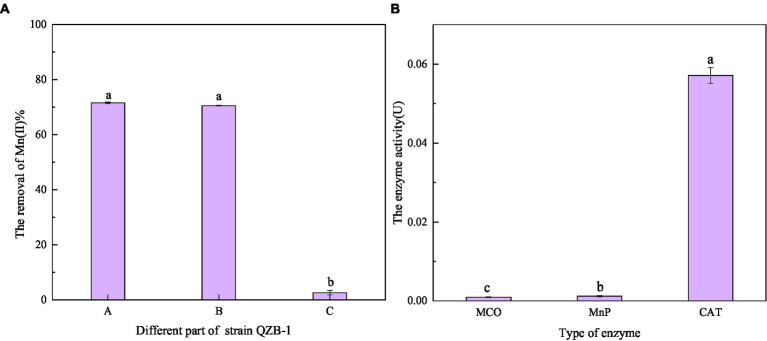
Oxidation ability of Mn(II) in different positions of strain QZB-1 **(A)**, and the enzymatic activity related to Mn(II) oxidation **(B)**. A: liquid supernatant; B: bacterial resuspension; C: bacterial lysate; MCO: multicopper oxidase; MnP: manganese peroxidase; CAT: manganese catalase.

As shown in Figure 7A, after reaction for 2 h, the Mn(II) oxidation efficiency of each part of strain QZB-1 was 71.56% (bacterial supernatant), 70.53% (bacterial resuspension), and 2.58% (bacterial lysate). The results suggested that strain QZB-1 could synthesize the Mn-oxidizing active factors within the cell and secrete them into the extracellular environment or cell surface to oxide Mn(II). This is consistent with the conclusion of previous studies (Yan et al., 2014; An et al., 2021). In addition, oxidative factors relative to the Mn oxidation by microorganisms were identified to be heme peroxidase (Anderson et al., 2009; Learman and Hansel, 2014), multicopper oxidase (Tang et al., 2019), and two-component regulatory protein (Geszvain et al., 2016). In this study, the activity of CAT was significantly (*p* < 0.01) higher than that of MnP and MCO (Figure 7B), indicating that CAT played a crucial role in the process of Mn(II) oxidation. Overall, strain QZB-1 could directly oxidize Mn(II) to Mn(IV) via Mn(III), which was catalyzed by Mn catalase released extracellularly. In addition, strain QZB-1 could also release alkaline substances to increase the pH of the culture medium, thus mediating the indirect oxidation of Mn(II).

#### 3.4.4. Changes in EPS composition

**Figure 8:**
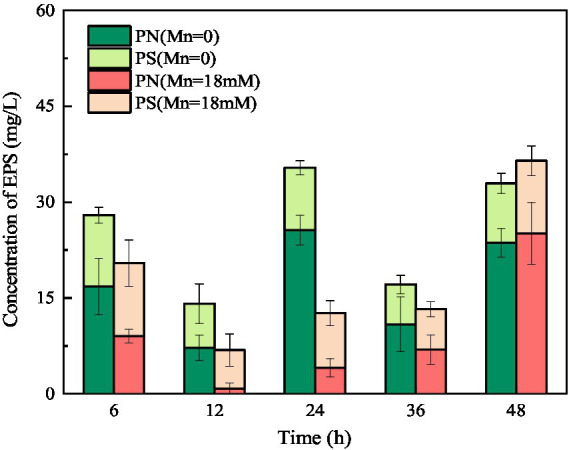
Changes in EPS concentration of strain QZB-1 during the cultivation process with 0 or 18 mM Mn(II).

Extracellular polymeric substances (containing PN and PS) play a key role in the biosorption of heavy metals owing to their active functional binding sites (Sheng et al., 2013). As shown in Figure 8, the amount of PN was consistently higher than that of PS in the culture medium without Mn(II), indicating that the main component of EPS secreted by strain QZB-1 was PN. Usually, microorganisms increase the production of EPS in the presence of heavy metals as a defense mechanism (Yue et al., 2015; Liu et al., 2017). On the contrary, the total amount of EPS secreted by strain QZB-1 in the medium with 18 mM Mn(II) was less than that in the medium without Mn(II) after 6–36 h of inoculation (Figure 8), further suggesting that 18 mM Mn(II) did not cause stress to the strain. Strain QZB-1 synthesized more PN but less PS under Mn(II) stimulation, consistent with the results of Bai et al. (2021). Proteins are excellent biosorbent for heavy metals (Mahapatra et al., 2020). Therefore, the increase in PN promoted the absorption of Mn(II) by strain QZB-1, thus realizing efficient removal of Mn(II).

The above results showed that strain QZB-1 was able to remove 98.4% of 18 mM Mn(II) in 48 h. Mn(II) removal characteristics experiment illustrated that QZB-1 possibly removes Mn(II) through adsorption and oxidation. The obtained data according to the SEM-EDS, XRD, FTIR, and XPS analyses, as well as the Mn(III) trapping experiment confirmed Mn(II) adsorption and oxidation processes in strain QZB-1. Furthermore, the results of the EPS experiment revealed that Mn(II) adsorption was mediated by PN secreted from strain QZB-1. Both direct and indirect Mn(II) oxidation processes exist in strain QZB-1. The direct oxidation of Mn(II) was catalyzed by strain M extracellularly released Mn catalase, while the increased culture pH mediated the indirect oxidation of Mn(II).

## 4. Conclusion

*Serratia marcescens* QZB-1, isolated from acidic red soil, can tolerate Mn(II) concentrations up to 364 mM and effectively remove 18 mM Mn(II) via adsorption and oxidation. Adsorption, which is low-cost, plays a dominant role in Mn(II) removal. Therefore, strain QZB-1 is a promising candidate for Mn(II) wastewater treatment.

## Data availability statement

The original contributions presented in the study are included in the article/Supplementary material, further inquiries can be directed to the corresponding author.

## Author contributions

XH: conceptualization, resources, data curation, visualization, and funding acquisition. XN and KL: methodology. KL and XH: software. XH and DJ: validation. YZ and PC: formal analysis. XN, KL, YZ, and PC: investigation. XH, XN, and YZ: writing—original draft preparation. XH, XN, KL, and DJ: writing—review and editing. XH and JX: supervision. DJ: project administration. All authors have read and agreed to the published version of the manuscript.

## Funding

This work was supported financially by the National Natural Science Fund of China (4210733), Natural Science Foundation of Guangxi (2022GXNSFBA035606), and China Postdoctoral Science Foundation (2022M710850).

## Conflict of interest

XH was employed by Guangxi Bossco Environmental Protection Technology Co., Ltd.

The remaining authors declare that the research was conducted in the absence of any commercial or financial relationships that could be construed as a potential conflict of interest.

## Publisher’s note

All claims expressed in this article are solely those of the authors and do not necessarily represent those of their affiliated organizations, or those of the publisher, the editors and the reviewers. Any product that may be evaluated in this article, or claim that may be made by its manufacturer, is not guaranteed or endorsed by the publisher.

## Supplementary material

The Supplementary material for this article can be found online at: https://www.frontiersin.org/articles/10.3389/fmicb.2023.1150849/full#supplementary-material
