# Supplementary figures and images for: Efficient Mn(II) removal mechanism by Serratia marcescens QZB-1 at high manganese concentration
Source: Front Microbiol. 2023 Apr 27;14:1150849. doi: 10.3389/fmicb.2023.1150849 (PMC10172493; doi:10.3389/fmicb.2023.1150849)

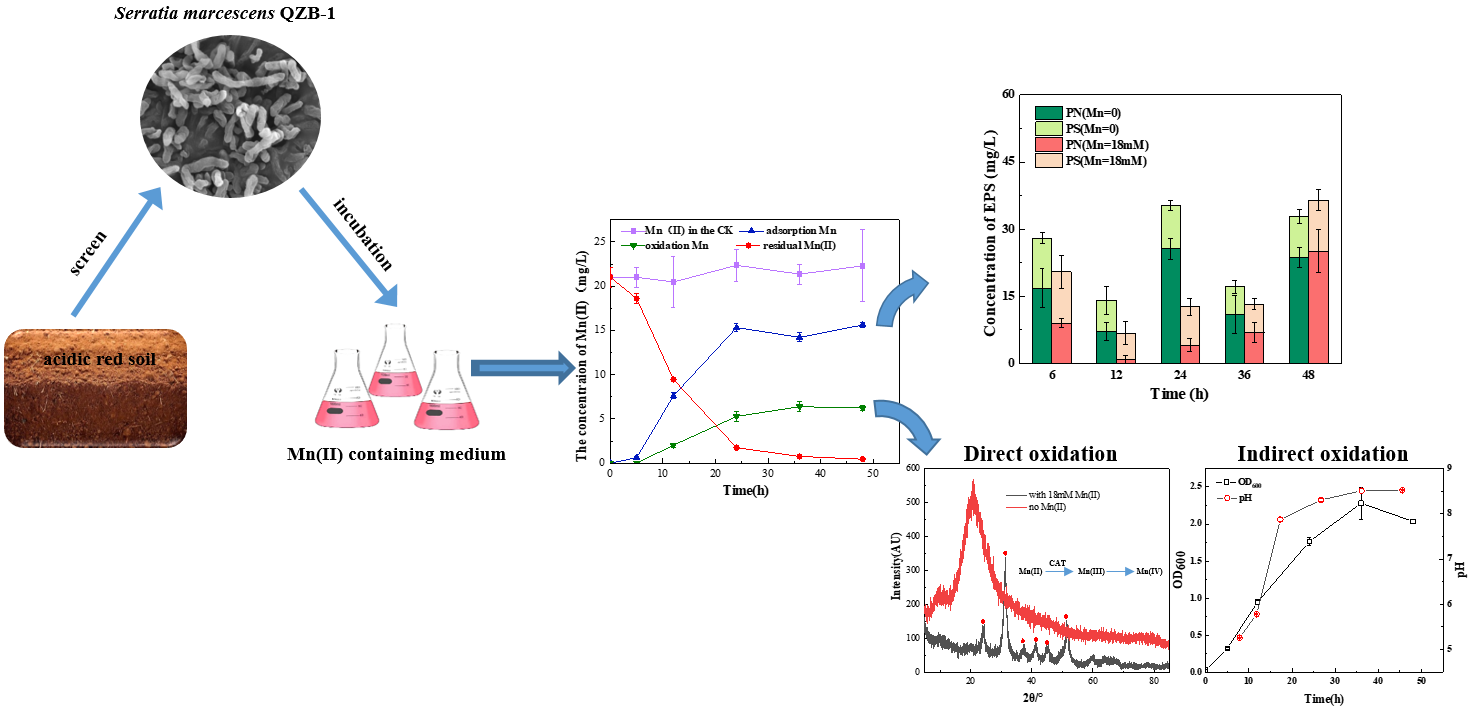

Supplement: Supplementary file 1 [file Data_Sheet_1.doc]
